# Supplementary material for: The interactome of intact mitochondria by cross-linking mass spectrometry provides evidence for coexisting respiratory supercomplexes
Source: Mol Cell Proteomics. 2017 Dec 8;17(2):216–32. doi: 10.1074/mcp.RA117.000470 (PMC5795388; doi:10.1074/mcp.RA117.000470)
Supplement: Supplemental Data [file supp_RA117.000470_133922_0_supp_23978_fzffwf.zip › spectra_annotation/mito_spectra_annotation/185-1-14-1-5-1.pdf]

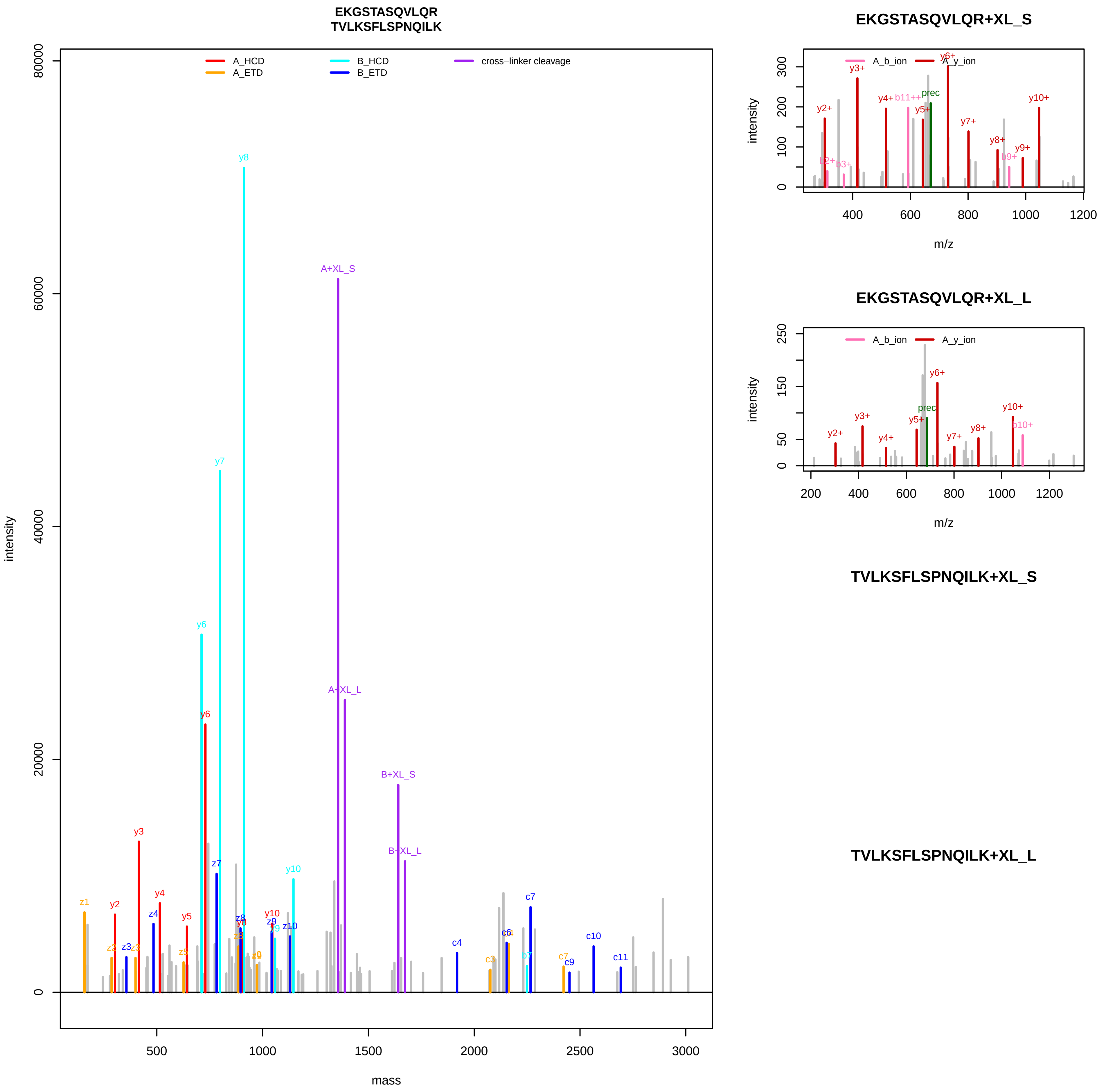
EKGSTASQVLQR
TVLKSFLSPNQILK
A_HCD
A_ETD
B_HCD
B_ETD
cross−linker cleavage
y8
A+XL_S
y7
y6
y6
A+XL_L
B+XL_S
B+XL_L
y3
y4
y2
z1
y5
z7
y10
z8
z9
z10
y9
c7
c4
c6
c3
c7
c9
c10
c11
z2
z3
z3
z4
z5
intensity
mass
0
20000
40000
60000
80000
500
1000
1500
2000
2500
3000
EKGSTASQVLQR+XL_S
A_b_ion
A_y_ion
y6+
y3+
y2+
y4+
b11++
prec
y5+
y10+
y7+
y8+
y9+
b9+
b2+
b3+
intensity
m/z
0
100
200
300
400
600
800
1000
1200
EKGSTASQVLQR+XL_L
A_b_ion
A_y_ion
y6+
prec
y3+
y5+
y2+
y4+
y7+
y8+
y10+
b10+
intensity
m/z
0
50
150
250
200
400
600
800
1000
1200
TVLKSFLSPNQILK+XL_S
TVLKSFLSPNQILK+XL_L
